# Assessment of interproximal enamel reduction planned by the digital set-up of a customized lingual orthodontic appliance: A comparison cohort study

**DOI:** 10.1016/j.heliyon.2024.e24361

**Published:** 2024-01-24

**Authors:** Jean-François Cuzin, Dominique Gaget, Petra Maes, Peter Bottenberg, Bart Vande Vannet, Karlien Asscherickx

**Affiliations:** a Private Practice, Nancy, France; b Private Practice, La tour du pin, France; c Université Lorraine, faculté d’Odontologie, département d'orthopédie dentofaciale, Nancy, France; d Université Libre de Bruxelles, Bruxelles, Belgium; e Vrije Universiteit Brussel, Brussel, België, Belgium

**Keywords:** Tooth size discrepancy (TSD), Interproximal enamel reduction, Virtual digital planning, Lingual orthodontic appliance

## Abstract

**Objectives:**

Interproximal enamel reduction (IER), commonly known as stripping, is a frequently used technique in orthodontic treatment to address issues related to arch length discrepancies and tooth size discrepancies (TSD). The use of digital set-up allows for precise prediction of the amount of IER required. TSD occurs when the sizes of maxillary and mandibular teeth are not in proportion to each other. This study aims to evaluate and compare the suggested IER values generated by the digital set-up of a customized lingual orthodontic appliance in both upper and lower arches, across sextants, and among different teeth concerning TSD.

**Materials and methods:**

We analyzed suggested IER values from 809 cases. The statistical analysis was divided into two parts: part 1 focused on the number of stripped surfaces, and part 2 assessed the quantity of enamel removed. Comparisons were made between upper and lower arches, sextants, and teeth using the Friedman test, followed by pairwise Wilcoxon tests with Bonferroni correction.

**Results:**

The study found that mandibular and frontal stripping were more frequently suggested than maxillary and posterior stripping. Lower canines were the teeth most commonly recommended for stripping, followed by upper incisors.

**Conclusion:**

Within the scope and limits of this cohort study, we conclude that, in general, more IER is required in the mandible as compared to the maxilla. Particularly in the anterior sextants, IER might be necessary to achieve optimal alignment and occlusion.

## 1 Introduction

The primary goal of comprehensive orthodontic treatment is to achieve an ideal functional occlusion, overbite, and overjet, while ensuring proper alignment of the teeth and a harmonious facial profile. However, the presence of arch length discrepancies (ALD) and/or tooth size discrepancies (TSD), can hinder the attainment of an optimal treatment outcomes. Studies have reported that significant tooth-size anterior discrepancies occur in 20–30 % of individuals, with 5–14 % presenting with overall TSD [1].

Proper occlusion, characterized by normal overjet and overbite, requires proportionality in the sizes of maxillary and mandibular teeth. Skeletal discrepancies often lead to dento-alveolar compensations, but it is crucial to position the teeth correctly within the available alveolar bone for a stable and well-aligned result. Expanding the dental arch to address crowding (ALD) issues may lead to instability over time [2].

TSD, characterized by disproportions in the size of individual teeth, plays a significant role in achieving proper occlusion with the correct overbite and overjet [[3], [4], [5]]. The mesiodistal widths of teeth, as originally investigated by Black, are still referenced in contemporary orthodontics [4,7].

Saatci and Yukay and Tong et al. have examined whether extracting four premolars as a requirement of orthodontic therapy contributes to TSD [5,6]. Pre-treatment mesiodistal dimensions of mandibular and maxillary teeth were measured, recorded on a computer program, and subjected to Bolton's analysis. They then performed hypothetical tooth extraction of all premolar combinations by a computer on each patient. Their results are in agreement with the opinion expressed by Bolton that the removal of the larger mandibular second premolars often improves the overall Bolton ratio. This factor is not large but may tip the balance in some extraction decisions [7,8].

Interproximal enamel reduction (IER) is a commonly used clinical orthodontic technique that allows for modification of the mesiodistal dimension of a tooth by elimination of a certain quantity of enamel via mechanical or manual action at the interdental contact points [[3], [4], [5], [6], [7]]. Interproximal enamel reduction has multiple objectives, including space-gaining by reduction of the mesiodistal tooth width for proper alignment of teeth in case of crowding, correction of tooth size discrepancies between upper and lower arch, or redirecting the contact point more apically to prevent esthetically unattractable black triangles [8,10]. IER has been suggested as a preventive and therapeutic measure and is a valuable technique g excellence in finishing orthodontic cases [[9], [10], [11]]. The fundamental changes in orthodontic practice, with emphasis on fewer extractions and greater importance on the stability of treatment [[10], [11], [12]], have made IER an integral part of orthodontic treatment.

Several IER methods have been tested and improved in recent years. The most used methods include manual stripping (hand-pulled strips), oscillating segmented discs, rotary discs and stripping discs with a polishing system (rotary instruments).

However, IER is not without risk factors, including hypersensitivity, potential damage to dental pulp, increased formation of plaque, the risk of caries in the stripped enamel areas, and periodontal issues [13,14].

However, when it is properly mastered, IER is a genuine therapeutic option for orthodontists [6], and negative outcomes can be avoided [15].

While numerous studies have discussed IER since the 1980s, most of them focused on various application techniques and the effects on enamel and the pulp chamber and only a few studies with a limited number of cases have discussed the extent of IER planned and executed during orthodontic treatment. Visual estimation (eyeballing) of TSD might not prove accurate, especially at the level of the bicuspid. Failure to make an accurate diagnosis often leads practitioners to face difficulties in the “finishing phase” when aligning the teeth correctly because of disproportionate maxillary-to- mandibular tooth sizes.

The aim of this study was to assess, analyze and compare the amount of IER required across arches, sextants, and teeth, by analyzing the IER values suggested in cases where a virtual set-up was made and the amount of IER necessary was calculated with the software of the “Harmony®” system (an individualized self-ligating lingual bracket system) in relation to the TSD.

The null hypothesis of this analysis is that there is no difference in the amount of IER necessary between arches, sextants, and teeth.

## 2 Materials and methods

We conducted a cohort study using IER data obtained from lingual set-up procedures performed by the Harmony® laboratory (American Orthodontics, Sheboygan, US) used for this study. The distribution of patients by country and year (between 2013 and 2017) was provided to the researchers in a Microsoft Excel workbook (IER lab Harmony participants stripping values). No further data like names, ages, gender, or treatment plan were provided. The identity of patients was not revealed to the researchers. The data were screened with approval from the medical ethics committee. The medical ethics committee of UZ Brussel, Brussels, Belgium had no ethical objections to the use of the aggregated data in this study. Registration number B.U.N. 143201838431.

**Fig. 1:**
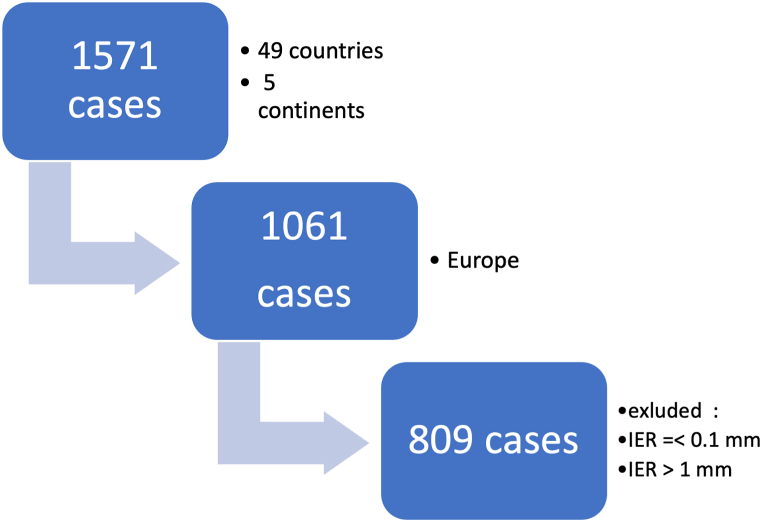
Flowchart presenting how data were delivered for analysis and how cases were selected.

Among the data provided by the Harmony® laboratory, only the data for the cases from Europe were selected for evaluation. Further exclusion criteria were IER values below 0.1 mm (technically unfeasible) and values exceeding 1 mm (clinically unacceptable due to the thinness of enamel) Enamel thickness might not even be 1 mm [16]. Fig. 1 depicts in a flow chart the case selection.

Values for IER were calculated with the proper software of the “Harmony®” system, to correct for arch length discrepancies and/or tooth size discrepancies to achieve a dental class I with proper overjet and overbite and a good alignment of the teeth. However, no data were available, on whether IER was first calculated for correction of ALD and afterward for TSD or vice versa. The practitioner could make suggestions for localization of IER according to the treatment goals and this would be considered when the virtual set-up was done. For example, in a case where there was a risk of development of black triangles after alignment, the practitioner could suggest IER in the upper front region. After the set-up was performed, the suggested treatment plan was sent to the practitioner for validation of the data before the finishing work (preparing individual brackets and wires) was done by the laboratory.

### 2.1 Statistical analysis

The statistical analyses of the suggested IER were performed in two parts as follows:

### 2.2 Part 1: statistical analyses by the number of stripped surfaces

Each surface to be stripped, regardless of the amount of removed tissue,was assigned a value of “1”, while non-stripped surfaces received a “0”. Summations were made for surfaces per jaw or sextant and statistical analyses were performed. Non-parametric methods were employed due to the categorical nature of the data(Y/N).

### 2.3 Part 2: statistical analyses by the quantity of enamel to be removed by IER

For the quantitative analyses (amount of tooth substance planned for a reduction in mm), only surfaces ≥0.1 mm were selected. If the amount was “0”, the surfaces were considered missing. Quantity was also added up per sextant or jaw.

Statistical and graphical calculations were performed using SPSS (IBM SPSS Statistics, Version 24.0. IBM Corp, Armonk, NY) and Graph Pad Prism software (Prism version 6.00 for Windows, GraphPad Software, La Jolla California USA). The statistical analyses were performed using non-parametric methods in multiple groups. Friedman tests were followed by Wilcoxon tests, with Bonferroni correction for paired data (within one case) or Kruskal-Wallis tests followed by pairwise Mann-Whitney tests with Bonferroni correction for unpaired data.

## 3 Results

The dataset encompassed 1571 cases of IER across 49 countries spanning five5 continents, with procedures performed between 2013 and 2017. Of these, 1061 cases originated from Europe. Following the application of exclusion criteria, 809 datasets were available for analysis (Fig. 1). Given the data on the amount of material to be removed via IER exhibited significant non-normal distribution (Shapiro-Wilk test, p < 0.01), statistical procedures were performed with nonparametric tests, and data displayed as median (IQR).

### 3.1 Analyses by jaw

**Fig. 2:**
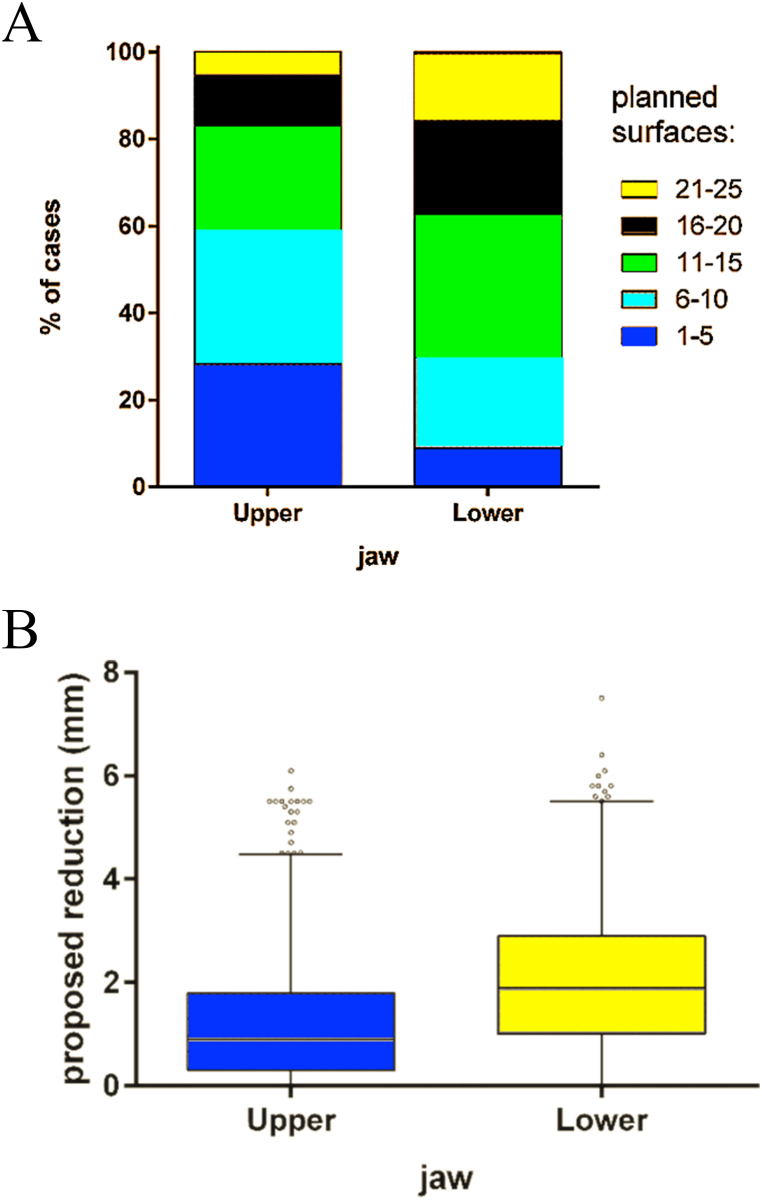
Percentage of cases with a certain number of surfaces scheduled for IER by jaw (A), the sum of proposed material to be removed by IER (mm) by jaw (B).

In the mandible, the number of surfaces earmarked for IER exceeded those in the maxilla (Wilcoxon test, p < 0.001) (Fig. 2A). Table 1 illustrates the percentage of cases based on the number of surfaces planned for reduction. In the maxilla, IER was required in 71.7 % of cases, with the majority (54.8 %) involving 1 to 10 surfaces. In the mandible, 91.1 % of cases required IER, primarily (75,5 %) within the range of 1–15 surfaces.

**Table 1:** Number of surfaces scheduled for IPR by jaw, expressed as % of cases.

	Number of surfaces planned for reduction (% of cases)					
	0	1–5	6–10	11–15	16–20	21–25
Maxilla	28.3	30.4	24.4	11.3	5.6	0.1
Mandibula	8.9	20.9	32.0	22.6	15.2	0.4

The cumulative proposed material reduction was significantly greater in the mandible as compared to the maxilla (Wilcoxon test, p < 0.001) (Fig. 2B). The average total suggested IER was 1.205 mm (range: 0–6.1 mm) in the maxilla and 2.096 mm (range: 0–7.5 mm) in the mandible.

### 3.2 Analyses by sextant

**Fig. 3:**
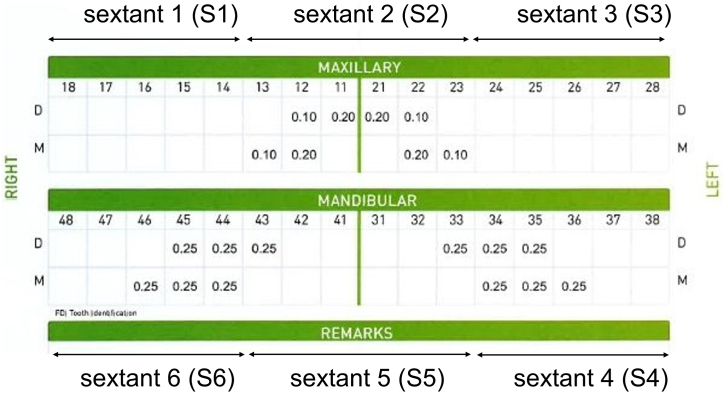
Chart of a case with proposed localization and amount of IER and schematic visualization of sextants. Sextant 1: right upper posterior sextant. Sextant 2: anterior upper sextant. Sextant 3: left upper posterior sextant. Sextant 4: left lower posterior sextant. Sextant 5: anterior lower sextant. Sextant 6: right lower posterior sextant.

This assessment aimed to evaluate the suggested IER differences among the three sectors per jaw (a total of six sectors): frontal canine-to-canine and lateral premolars and molars. Fig. 3 presents a chart of a case, where IER is proposed, along with indicated sextants.

Table 2 depicts the results of the data analysis per sextant concerning the number of surfaces planned for IER.

**Table 2:** Number of surfaces scheduled for IPR by sextant, expressed as % of cases.

	number of surfaces planned (% of cases)		
Sextant	0	1–5	6–10
S1	83.1	16.9	0
S2	34.7	41.2	24.1
S3	84.3	15.7	0
S4	58.2	41.8	0
S5	20.4	37.5	42.2
S6	58.3	41.7	0

**Fig. 4:**
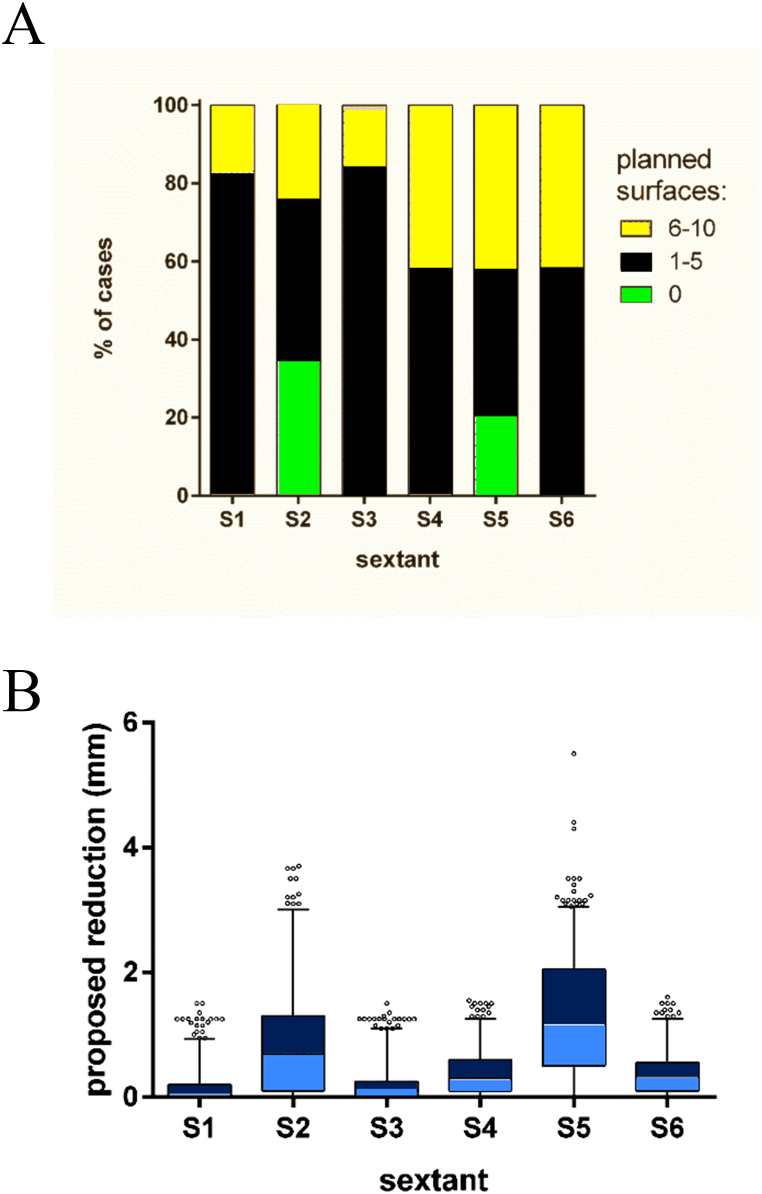
Percentage of cases with a certain number of surfaces scheduled for IER by sextant (A), the sum of proposed material to be removed by IER (mm) by sextant (B).

The lower frontal sextant (S5) exhibited the highest frequency of surfaces slated for IER (79.6 %), significant different from the upper frontal sextant (S2) (65.3 %) and both lower posterior sections (S4 and S6). The frequency of proposed IER in the upper frontal sextant was significantly higher than in left or right posterior sextants (S1 and S3). No significant differences were found between right or left posterior sextants when observing a single jaw. Both the frequency and amount of IER proposed were significantly higher in the lower posterior sextants as compared to the upper posterior sextants. These findings apply as well for the number of planned surfaces as for the quantity of material to be removed (Fig. 4A and B).

Table 3 provides the results of the data analysis per sextant when the amount of material planned for IER is considered. In the frontal sextants, more material was scheduled to be removed with on average 0.8716 mm (range 0–3.7 mm) in sextant 2 and 1.323 mm (range 0–5.5 mm) in sextant 5.

**Table 3:** Descriptive statistics of the amount of material proposed for IPR (mm) by sextant.

Sextant	1	2	3	4	5	6
Mean	0.165	0.8716	0.1683	0.3923	1.323	0.3804
Median	0.0	1.3	0.0	0.3	1.2	0.3
Minimum	0	0	0	0	0	0
Maximum	1.5	3.7	1.5	1.55	5.5	1.6

Table 4 illustrates post hoc pairwise comparisons between sextants.

**Table 4:**
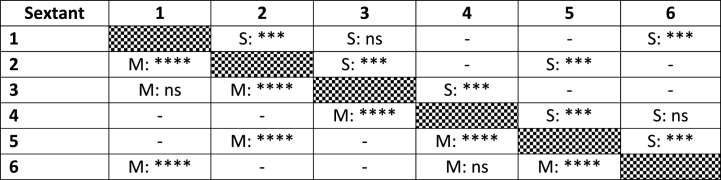
Results of Friedman related data ANOVA followed by Bonferroni-corrected pairwise comparisons between sextants. Upper part: number of surfaces (S), lower part: quantity of material (M). ns: not significant, ***: p < 0.001, ****: p < 0.0001, -: no comparison performed to avoid spurious results.

## 4 Analyses based on tooth number

**Fig. 5:**
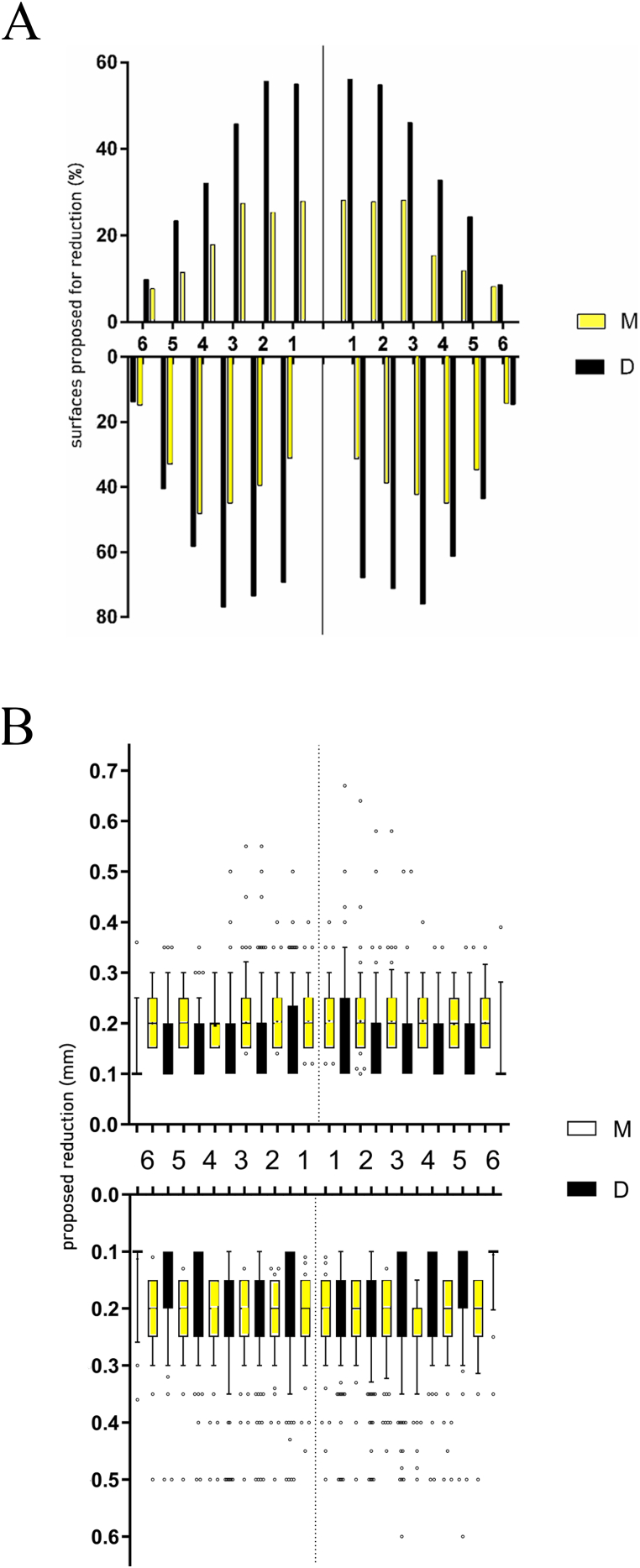
Percentage of cases with a certain number of surfaces scheduled for IER by tooth (FDI notation) and by surface (mesial/distal) (A), proposed material to be removed by IER (mm) by tooth (FDI notation) and surface (mesial/distal) (B).

In the mandible, the teeth with the highest frequency of planned IER were the lower canines. In the lower right canine (tooth number 43) at least one stripped surface was suggested in 83.9 % of the cases and lower left canine (tooth number 33) 82 % of cases where IER was suggested for at least one surface. In the maxilla, the teeth with the highest frequency of planned IER were the incisors. The lowest number of planned surfaces were found on the upper molars (ca 16 %) and lower molars (ca 28 %). All teeth exhibited a significantly larger number of stripped surfaces (Paired Wilcoxon test, p < 0.05) on the distal side, except for teeth 16, 26, 36, 35, 45, and 46 (Fig. 5A). However, the material suggested to be removed was significantly (p < 0.05) higher in mesial compared to distal side except for all molars and mandibular canines (Fig. 5B).

Based on the results of the analyses, the null hypothesis that there is no difference in the amount of IER necessary between arches, sextants, and teeth must be rejected.

## 5 Discussion

Interproximal enamel reduction (IER) is a crucial aspect of orthodontic treatment, particularly in the finishing stage, where practitioners often face challenges in achieving both perfect occlusion posteriorly and ideal alignment anteriorly. Frequently, tooth size discrepancies are present hindering the attainment of optimal occlusion and alignment, without interproximal enamel reduction. Understanding how frequently IER is required for routine orthodontic treatment and where it is most needed can aid practitioners in treatment planning.

The aim of this study was to perform a statistical analysis of the suggested stripping values, generated by the digital set-up of a customized lingual orthodontic appliance known as“Harmony®” across upper and lower arches, between sextants, and between different teeth.

The values of suggested IER resulted from the virtual set-up, primarly conducted by the laboratory, sometimes after input of the practitioner, suggesting specific IER sites to the laboratory. Practitioner input typically focused on aesthetics (avoidance of black triangles after alignment) while the input of the virtual set-up would relate to the achievement of a good occlusion and alignment. Virtual set-up considers tooth size ratios, upper incisor thickness, anterior incisor inclination and torque, overjet, and overbite.

It is important to noted that in this study analyzed data from the planning software, which had been validated by the dental practitioners. However, it cannot be confirmed that all the planned and/or proposed stripping protocols were executed as intended in all patients [16,17]. Furthermore, there may a difference between the planned and achieved tooth reduction. This has been described in a study by Kalemaj and Levrini, indicating that implemented IER seemed to be less than planned IER, especially for lower canines and distal surfaces of teeth [18]. De Felice and colleagues concluded that the amount of enamel removed in vivo did not correspond with the amount of IER planned [19]. In most cases, the performed IER amount was lower than planned. On the other hand Lagana and colleagues found in their study, that the amount of enamel removed in vivo corresponded with the amount of IER planned by the orthodontist [20]. The more accurate results of the latter study might be explained by the stripping method. While in the study of Kalemaj and Levrini different methods were used for IER, in the study of Lagana and colleagues the only stripping method used was a mechanical oscillating system [17,21].

The results of the this study suggest that more IER is necessary for the mandible as compared to the maxilla. This aligns with findings from two other studies, albeit those studies had limited case numbers. One study on Invisalign® therapy (30 patients) reported planned IER in the maxilla of 0.62 mm on average (range 0.20–2.20 mm) and in the mandible of 1.92 mm on average (range: 0.20–4.40 mm) [22]. In another study with clear aligner treatment (40 patients) planned IER was 1.09 mm on average in the maxilla (SD 1.13 mm, range 0.30–3.60 mm) and 1.43 mm on average in the mandible (SD 1.10 mm, range 0.40–3.50 mm) [14]. While these two studies were conducted with a limited number of cases, our study with many cases confirmed the results found in the other studies.

Different reasons might explain why more IER is planned in the mandible as compared to the maxilla. In general orthodontic cases, crowding is more pronounced in the mandible as compared to the maxilla. Class II camouflage treatment is frequently performed and due to the compensatory tipping of maxillary and mandibular incisors, the anterior arch length ratio is negatively influenced, necessitating IER in the mandible [24].

Tooth size discrepancy consists of two components an Anterior Ratio and an Overall Ratio. In the anterior region, upper lateral incisors are often too small. In the posterior region, upper bicuspids might be smaller than lower bicuspids. This was confirmed in a study where the effect on the Bolton value was evaluated if four first bicuspids would be extracted or another combination of bicuspid extractions. It appeared that upon extraction of four first bicuspids, more frequent and greater discrepancies were created, indicating a major TSD between lower and upper second bicuspids. A clinically significant tooth-size discrepancy was observed in 32 patients out of 50 patients. Whereas other combinations with the extraction of second lower bicuspids, decreased the TSD, because of the wider mesiodistal dimension of the second lower bicuspid [25].

Begg's specific figure of a 10.54 mm loss of mandibular tooth material (in the mesiodistal axis, because of attrition before age 18) has been widely repeated in the anthropologic and orthodontic literature [[23], [24], [25]]. Our comparative cohort study shows a much smaller amount of interproximal enamel reduction respectively 1.205 mm (range: 0–6.1 mm) in the maxilla and 2.096 mm (range: 0–0.75 mm) in the mandible. Our observation could be in relation to the calculated overall and anterior ratio.

The analyses by sextant were performed to be able to make a comparison of IER needed between anterior and posterior segments. More IER was suggested in the frontal sextants as compared to the posterior sextants. There might be different reasons. Probably one reason is to avoid the development of black triangles after alignment. The appearance of the maxillary incisors is a major aesthetic criterion as is the appearance of the mandibular incisors. Frontal mandibular crowding tends to increase with age due to the physiological migration of the lateral-posterior sectors (secondary migration). While no data were available concerning the age of the patients, since it concerns a lingual technique, it can be assumed that most patients were adults; however, this could not be substantiated by the data at hand. In cases with dentoalveolar compensation, the treatment goal may be to resolve anterior crowding without altering posterior occlusion. Another reason for more IER planned in the anterior region, might be that IER is more straightforward to perform there compared to the posterior section.

The teeth most often suggested for IER in the present study were the incisors in the maxilla and the canines in the mandible. The choice for IER in the upper front probably was likely made to address crowding, prevent flaring of the teeth and avoid black triangles. The lower canines were the teeth most often planned for IER in the mandible. The proximal enamel thickness (PET) is typically larger in the canines as compared to the incisors in the mandible, which might explain the preference for stripping canines rather than incisors to address crowding [20]. While the distal side of a tooth was more often suggested for IPR, the amount of material to be removed was larger on the mesial side. No specific explanation for this finding was identified.

Due to data and privacy protection, these retrospective data could not be linked to patient data. Consequently, more detailed, such as linking suggested IER requirements to differences in Angle Class malocclusions, age, gender and other factors, where not feasible. This explains why in the analyses, no distinction could be made between the amount of IER which was necessary for the correction of arch length discrepancy (ALD), and the amount of IER which was necessary to correct for TSD. This is a big limitation of this study. Prospective studies where patients diagnoses and treatment plans are provided to researchers with patient consent, may enable the exploration of such relationships.

Nevertheless, the extensive dataset offers valuable insights into the location and extent of IER commonly required orthodontic cases to achieve favorable aesthetic and occlusal outcome. The choice of where and how much IER to perform, considering practitioners input and execution of the Harmony® set-up) remains at the discretion of the practitioner.

The virtual set-up of a case gives the ability to predict discrepancies before initiating treatment. Tooth size ratios may be influenced by other factors such as upper incisor thickness, anterior incisor inclination, overjet, and overbite [22]. The virtual set-up allows the orthodontist to adjust the treatment plan and provides the most efficient and effective way to help the patient.

The analyses in this study were performed on data of virtual set-up for an individualized lingual bracket system, but conclusions are valuable for every orthodontic treatment, be it lingual orthodontics, vestibular orthodontics, or aligner therapy. Before starting orthodontic treatment, patients should be warned that a certain amount of interproximal enamel reduction might be necessary, to achieve good alignment and occlusion. Indication, of where this will be performed, can be derived from this study.

## 6 Conclusions

Based on the analyses of 809 data sets, from the Harmony® laboratory, and within the limits of this study, the following conclusions can be drawn:

- Mandibular IER is more frequently required compared to maxillary IER, both in terms of the number of stripped surfaces and the quantity of material to be removed.
- The mandibular frontal sextant is the area where IER is most often necessary, followed by the maxillary frontal sextant.
- The teeth most often planned for IER are the lower canines, followed by the upper incisors.

## 7 Limitations and strengths

Since no details of treatment plan per case were available, it was not possible to determine how much of the IER was needed for ALD and how much for TSD. This is a limitation of the study.

A significant, strength of the study lies in the large number of cases evaluated. Some outcome variables exhibited significant differences, demonstrating sufficient statistical power for these variable-test combinations.

Another strength is that results can be extrapolated to any kind of appliance used, be it lingual bracket system, buccal bracket system or aligner therapy.

## Data availability

Data available on request.

## Ethical committee information

Upon review of the documents that were submitted to the medical ethics committee, and based on the responses and additional information that was provided by the investigator, the medical ethics committee UZ Brussel/VUB has no ethical objections to the use of the aggregated data in this study. Registration number B.U.N. 143201838431.

## CRediT authorship contribution statement

**Jean-François Cuzin:** Writing – original draft, Resources, Investigation, Data curation, Conceptualization. **Dominique Gaget:** Writing – original draft, Resources, Data curation, Conceptualization. **Petra Maes:** Supervision. **Peter Bottenberg:** Writing – original draft, Methodology, Formal analysis, Data curation. **Bart Vande Vannet:** Writing – review & editing, Visualization, Supervision, Resources, Project administration, Methodology, Funding acquisition, Formal analysis, Conceptualization. **Karlien Asscherickx:** Writing – review & editing, Writing – original draft, Visualization, Validation, Supervision, Project administration, Methodology, Formal analysis.

## Declaration of competing interest

The authors declare the following financial interests/personal relationships which may be considered as potential competing interests:Bart Vande Vannet reports financial support was provided by American Orthodontics. Bart Vande Vannet reports a relationship with American Orthodontics that includes: funding grants. If there are other authors, they declare that they have no known competing financial interests or personal relationships that could have appeared to influence the work reported in this paper.

## Abbreviations

IER: interproximal enamel reduction
Mm: millimeters
S: surfaces
M: material
FDI code: FDI World Dental Federation notation
TSD: tooth size discrepancy
ALD: arch length discrepancy
